# Selective repression of *RET* proto-oncogene in medullary thyroid carcinoma by a natural alkaloid berberine

**DOI:** 10.1186/s12885-015-1610-5

**Published:** 2015-08-26

**Authors:** Vishnu Muthuraj Kumarasamy, Yoon-Joo Shin, John White, Daekyu Sun

**Affiliations:** 1 College of Pharmacy, University of Arizona, Tucson, Arizona 85721; 2 BIO5 Institute, 1657 E. Helen Street, Tucson, Arizona 85721; 3 Arizona Cancer Center, 1515 N. Campbell Avenue, Tucson, Arizona 85724

## Abstract

**Background:**

The gain-of-function mutation of the *RET* proto-oncogene, which encodes a receptor tyrosine kinase, is strongly associated with the development of several medullary thyroid carcinomas (MTCs). Thus, the RET protein has been explored as an excellent target for progressive and advanced MTC. In this study we have demonstrated a therapeutic strategy for MTC by suppressing the transcription of *RET* proto-oncogene though the stabilization of G-quadruplex structure formed on the promoter region of this gene using a natural product berberine.

**Methods:**

Medullary thyroid carcinoma (MTC) TT cell line has been used to evaluate the effects of berberine on RET expression and its downstream signaling pathways. The specificity of berberine was demonstrated by using the papillary thyroid carcinoma TPC1 cell line, which lacks the G-quadruplex forming sequence on the *RET* promoter region due to chromosomal rearrangement.

**Results:**

Berberine suppressed the RET expression by more than 90 % in MTC TT cells at a concentration of 2.5 μg/ml with minimal effect on the TPC1 cells. Canadine, which is a structural analogue of berberine, showed little interaction with RET G-quadruplex and also had no effect on RET expression in MTC TT cells. The down-regulation of RET with berberine further inhibited the cell proliferation through cell cycle arrest and activation of apoptosis in TT cells, which was confirmed by a 2-fold increase in the caspase-3 activity and the down-regulation of cell-cycle regulatory proteins.

**Conclusion:**

Our data strongly suggest that the G-quadruplex forming region and the stabilization of this structure play a critical role in mediating the repressive effect of berberine on *RET* transcription.

## Background

Medullary Thyroid Carcinoma (MTC), which originates from the parafollicular C cells of the thyroid gland, accounts for 5 % of all thyroid malignancies [1]. The MTCs are either sporadic or inherited by a germline point mutation in the *RET* (REarranged during transfection) proto-oncogene as a part of multiple endocrine neoplasia type 2 (MEN2) syndrome [2, 3]. The *RET* proto-oncogene encodes a tyrosine kinase receptor on the cell surface, which binds the members of the glial cell line-derived neurotrophic factor (GDNF) family of ligands via the ligand binding site on the extra-cellular domain [4, 5]. This results in receptor activation through the dimerization of RET and trans-autophosphorylation of the tyrosine residues within the intra-cellular domain [6]. The activated RET triggers a cascade of internal signaling pathways, which include the MEK/ERK and PI3K/Akt that are responsible for cell proliferation and survival respectively [7–10]. In MEN2, a point mutation in one of the cysteine residues of the intracellular domain confers a ligand-independent dimerization of the receptor, thereby leading to constitutive activation of the mitogenic signaling pathways [11, 12]. In accordance with the pivotal role of RET in regulating the growth of thyroid cancer cells in tumor development, there are clinically available drugs for MTC therapy such as cabozantinib and vandetanib that inhibit the tyrosine kinase activity of RET [13–16]. Because of their potential inhibitory effects on other tyrosine kinase receptors like VEGFR, EGFR, MET etc. [15, 16], the development of a specific RET kinase inhibitor still remains challenging. Moreover, the development of resistance against the kinase inhibitors has been reported in MTC due to the mutations at gatekeeper residues, which render the receptor inaccessible to drug binding [17, 18]. To overcome these challenges, we have been developing a new strategy to turn off the transcription of *RET* proto-oncogene using small molecules rather than directly inhibiting the kinase activity of the receptor [19].

Our previous study has revealed that the G-rich sequence of the polypurine/polypyrimidine tract in the proximal promoter region of the *RET* gene is structurally dynamic in nature [20]. This facilitates the conformational transitions between the B-DNA and higher-order secondary structures like G-quadruplexes under the influence of negative supercoiling [21, 22]. The formation of G-quadruplexes involves the stacking of two or more G-tetrads over each other and each G-tetrad comprises a square planar arrangement of four guanine residues joined together through Hoogsteen hydrogen bonding [23–25]. The biological relevance of G-quadruplexes is well established based on their formation in the telomere region and also in the promoter region of other oncogenes [22–26].

Furthermore, we have demonstrated that the G-quadruplex structure is involved in down-regulating the transcription of *RET* gene and that could also be controlled by the stabilization of this structure using small molecules [19]. In order to discover more drug-like molecules for our current study that exhibit transcriptional inhibitory effect on the *RET* gene, we searched for compounds that potentially stabilize the G-quadruplex structures *in vitro*. To date, several small molecules have been characterized as G-quadruplex binding agents; and among them, berberine is one of the most potential compounds to stabilize these structures formed in the telomere region and also in the promoter region of *c-Myc* oncogene [27–30]. Berberine is a natural alkaloid found in the Chinese herbal plants [31] which has an N^+^-containing isoquinoline aromatic ring with a planar conformation that allows π-stacking interactions with a tetrad in the G-quadruplex structure [27, 28].

In this study, we investigated the interaction of berberine with G-quadruplex structure formed on the *RET* promoter region using various biophysical and biochemical methods. We also explored the cellular effects mediated by berberine in MTC TT cells to gain further insight in developing berberine as a potential therapeutic agent for MTC treatment by suppressing the *RET* transcription.

## Methods

### Chemicals

Berberine was obtained from Sigma Genosys (Woodlands, TX, USA). Canadine and other compounds were kindly provided from the DPT (NCI/NIH). All the compounds were dissolved in DMSO at a final concentration of 10 mg/mL.

### Materials

The 5′-FAM labelled RET-WT (5′-AGCGGGTAGGGGCGGGGCGGGGCGGGGGCGG- 3′) and RET-MT1 (5′- AGCGGGTAGGAGCGGAGCGGGGCGGGGGCGG-3′) oligos were purchased from Sigma Genosys (Woodlands, TX, USA). Taq DNA polymerase was purchased from Fermentas (Hanover, MD, USA).

### Cell culture & media

The human medullary thyroid carcinoma cell line (TT) was obtained from the American Type Culture Collection (Manassas, VA, USA) and it was cultured in DMEM 1640 medium (Cellgro, Manassas, VA, USA) supplemented with 15 % heat inactivated fetal bovine serum (FBS). The isogenic cell lines HEK293-WT and HEK293-MT1, which have the luciferase reporter gene under the control of wild type and G4 KO (harboring two point mutations on the GC box region) *RET* promoters respectively were generated as described in our previous study (19). They were cultured in DMEM 1640 medium supplemented with 9 % FBS. The human papillary thyroid carcinoma cell line (TPC1) was received from Dr. Rebecca Schweppe (University of Colorado, Denver) and was cultured in RPMI-1640 medium supplemented with 9 % FBS. All the cell lines were maintained in a humidified atmosphere containing 5 % CO_2_ at 37 °C. The stocks for all the cell lines were obtained from the cell bank and were used within 6 months. We were also informed that these cell lines were mycoplasma free and were further authenticated using STR profiling.

### Polymerase stop assay

**Fig. 1:**
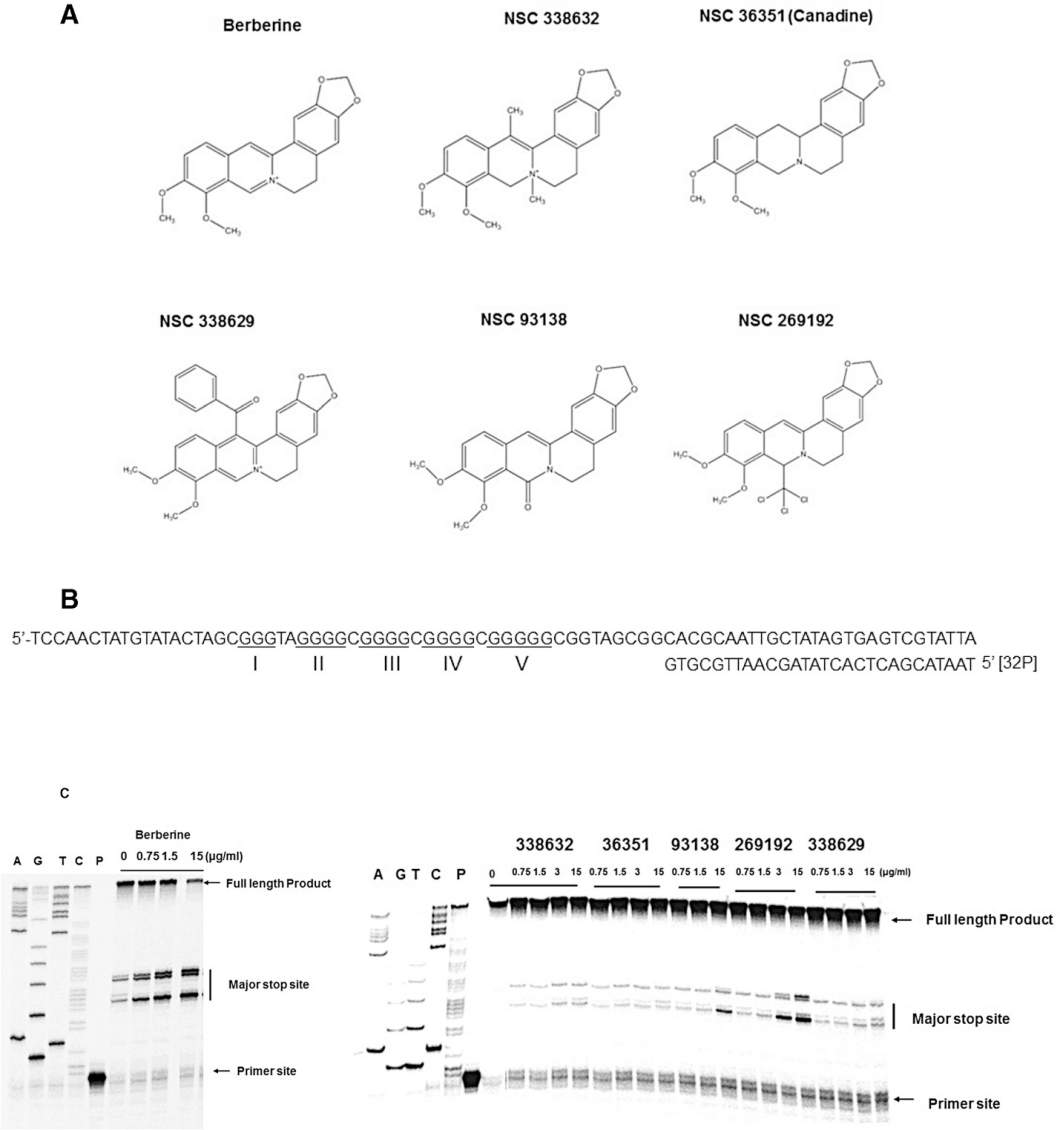
Taq DNA polymerase stop assay showing the stabilization of the RET G-quadruplex with berberine and its structural analogues. **a** Structures of berberine and its analogues. **b** Sequence of the single-stranded template DNA annealed with the 5′-[^32^P] labelled primer used in DNA polymerase stop assay. **c** DNA polymerase stop assay at increasing concentrations of berberine (0, 0.75, 1.5, 15 μg/ml) and its analogues (0, 0.75, 1.5,3, 15 μg/ml). Lanes A, G, T & C represent the di-deoxy sequencing reactions with the same template, which serve as the marker to identify the exact stop site. Lane P represents the position of the free primer on the gel

The 5′-end labeling with [γ]-^32^P and the DNA polymerase assay were performed as described previously [19, 20, 32]. The template DNA containing the G-quadruplex forming sequence present at the upstream of *RET* promoter region (−51 to −33 relative to transcription start site) were annealed with the primer (P28) labeled with [γ]-^32^P (Fig. 1b). The primer-annealed DNA templates were separated from excess labeled primer or remaining template DNA on 8 % non-denaturing polyacrylamide gel followed by gel-purification and used in a primer extension assay by Taq DNA polymerase.

### Dimethyl Sulfate (DMS) foot printing

The DMS foot-printing with the 5′-FAM-labelled RET-WT and RET-MT1 oligonucleotides were performed as described in our previous study [19]. In brief, the oligonucleotides that were folded into the G-quadruplex conformation were treated with DMS (0.2 %) for 2 min and were then resolved on a 16 % non-denaturing gel. Each DNA band was recovered from the gel and treated with piperidine (10 %) following ethanol precipitation. The cleaved products were resolved on a 16 % denaturing PAGE along with the sequencing markers. The gel was dried and the fluorescence was read on a Typhoon 8600 scanner (GE Heathcare Life Sciences, Pittsburgh, PA, USA) for analysis.

### CD spectroscopy

The RET WT oligonucleotide was diluted to a strand concentration of 5 μM in the presence of Tris–HCl buffer (20 mM, pH 7.6) and 25 mM KCl and allowed to form G-quadruplex structure by denaturing at 95 °C for 5 min and slowly cooled to room temperature. The temperature/wavelength CD spectra were recorded using a Jasco-810 spectrophotometer (Easton, MD, USA) using a quartz cell of 1 mm path length and an instrument scanning speed of 100 nm/min with a response time of 1 s over a wavelength range of 230–330 nm at increasing temperatures from 20 °C to 90 °C. Tm was determined by monitoring the molar ellipticity versus temperature profiles at 262 nm using a temperature gradient of 1 °C/min from 20 to 90 °C as described previously [19].

### Reverse Transcription PCR analysis of the RET mRNA synthesis in TT cells

The mRNA expression in TT cells following 24 and 48 h treatments with increasing concentrations of berberine was determined by RT-PCR as described previously [19]. The primers used for RT-PCR were as follows: RET forward: (5′-GCAGCATTGTTGGGGGACA-3′) and RET reverse (5′-CACCGGAAGAGGAGTAGCTG-30); Rpl9 forward (5′ CTGAAGGGACGCACAGTTAT-3′) and Rpl9 reverse (5′- ACGGTAGCCAGTTCCTTTCT-3′). The RET/PTC1 expression was determined by using the primers RET/PTC1 forward (5′- GTCGGGGGGCATTGTCATCT-3′) and RET/PTC1 reverse (5′-AAGTTCTTCCGAGGGAATTC-3′) as described before [33]. The PCR reactions involved an initial denaturation at 95 °C for 3 min followed by 33, 35 and 23 cycles for RET, RET/PTC1 and Rpl9 respectively, at 95 °C for 30 s, 52 °C for 30 s and 72 °C for 30 s on a GeneAmp PCR system 9600 (Perkin-Elmer, Waltham, MA, USA). The PCR products were analyzed on 1.5 % agarose gel electrophoresis.

### Western blotting

The whole-cell extracts were prepared and the proteins were resolved on a 4-12 % gradient polyacrylamide SDS-PAGE, as described previously [19]. The primary antibodies used were as follows: anti-RET (# 3220), anti-pAkt (# 9271), anti-Akt (# 9272) and anti-p-pRb (# 9307S) were purchased from Cell signaling, Beverly, MA, USA, anti-VEGF (sc-152), anti-Bcl-2 (sc-509), anti-c-Myc (product sc-40), anti-pERK1/2 (sc-81492), anti-ERK1/2 (sc-271270), anti-pMEK1/2 (sc-81503), anti-MEK1/2 (sc-81504), anti-E2F1 (sc-56661), anti-cyclin E (sc-247) and anti-pRb (sc-50) were purchased from Santa Cruz Biotechnology, and anti-actin (ab8227) was purchased from Abcam. Mouse or rabbit IgG antibodies conjugated with horseradish peroxidase (HRP) (BioRad) were used as secondary antibodies. An enhanced chemiluminescence kit (Santa Cruz Biotechnology) was used for the detection of the luminescence.

### Luciferase assay

The isogenic cell lines HEK293-WT and HEK293-MT1 were treated with different concentrations of berberine following 24 h exposure. Luciferase expression level is determined following the exposure to berberine using the ONE-Glo Luciferase Assay System (Promega) following the manufacturer’s instruction.

### Chromatin Immuno-precipitation Assay (ChIP)

CHIP assay was performed to determine the binding of SP1 and RNA pol II to the *RET* promoter region using the EZ-CHIP^TM^ (Millipore) as described previously [19]. The immunoprecipitation was performed using monoclonal mouse anti-SP1 (Sigma) and monoclonal mouse anti-RNA Pol II. The binding of SP1 and RNA pol II were quantified using RET promoter forward primer (5′- AAGCCCCACCCGGCCCAAG-3′) and reverse primer (5′-GACGGACACTGGGGGCGCGA-3′). The PCR reactions involved an initial denaturation at 95 °C for 3 min followed by 40 cycles at 95 °C for 30 s, 52 °C for 30 s and 72 °C for 30 s as described previously. The PCR products were analyzed on 2 % agarose-gel electrophoresis.

### Cell viability assay

Cells were plated at a concentration of 7500 cells/well in a 96-well plate and incubated overnight, followed by the exposure to the compounds at wide range of concentrations (0 – 6.25 μg/ml) up to 24, 48 and 96 h. The cell viability was determined by using 1 mg/ml MTT dye as described previously [34]. The absorbance was measured at 590 nm using a Synergy HT Multi-detection Microplate Reader (BioTek, Winooski, VT, USA).

### Caspase-3 assay & flow cytometry

TT cells were treated with different concentrations of berberine up to 48 h and caspase-3 activity was measured using the ApoAlert Caspase Fluorescent Assay kit (Clonetech Laboratory, Mountain view, CA, USA) by following the manufacturer’s protocol. FACS analysis was performed in TT cells following the treatment with different concentrations of berberine up to 48 h, as described in our previous report [19].

## Results

### Interaction of berberine with the RET G-quadruplex *in vitro*

In this study, we first examined the interactions of berberine and its structural analogues (Fig. 1a) with the RET G-quadruplex using DNA polymerase stop assay. In this assay, the formation of secondary structures like the G-quadruplex on the template DNA or the ligand-mediated stabilization of these structures is expected to arrest the progression of Taq-DNA polymerase during primer extension [32]. As shown in Fig. 1c, a dose-dependent increase in the amount of arrested products was observed in the presence of berberine, indicating the strongest interaction of this compound among its analogues with the RET G-quadruplex.

**Fig. 2:**
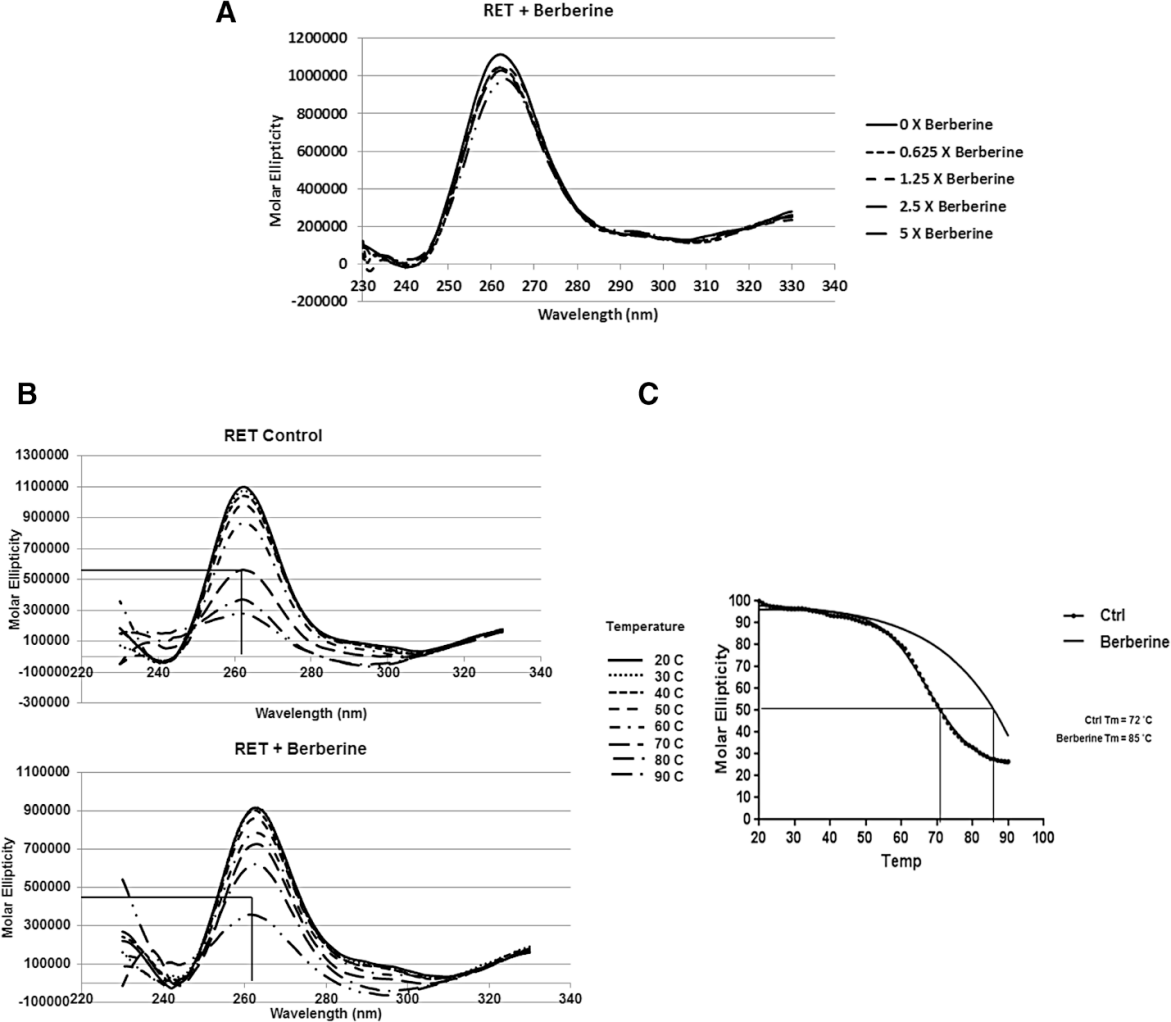
CD spectroscopic studies to validate the stabilization of RET G-quadruplex with berberine. **a** CD titration spectra for the RET-WT (5 μM) in the absence and presence of increasing concentrations of berberine (**b**) CD titration spectra for the RET-WT (5 μM) in the absence and in the presence of berberine (5 Equivalents) with increasing temperatures from 20 °C to 90 °C. **c** Melting curve of the RET-WT G-quadruplex in the absence and in the presence of berberine (5 Equivalents)

To further validate the stabilization of the RET G-quadruplex with berberine, CD spectroscopic analysis was performed for the RET-WT sequence in the absence and presence of berberine. First, we determined whether the binding of berberine changes the structural configuration of the RET G-quadruplex structure by monitoring the CD spectra in the absence and presence of increasing concentrations of berberine. As shown in the Fig. 2a the titration of RET-WT sequence (5 μM) with increasing concentrations of berberine did not affect the peak at 262 nm suggesting that berberine does not alter the parallel configuration of the RET G-quadruplex structure [35]. Next, we determined whether the binding of berberine stabilizes the G-quadruplex structure by monitoring the CD spectra in the absence and presence of berberine (5 Equivalents) at increasing temperatures. As shown in Fig. 2b, the intensity of the maximum peak at 262 nm gradually decreased as the temperature increases from 20 °C to 90 °C and a 50 % decrease was observed at ~72 °C, which corresponds to its melting temperature (T_m_) (Fig. 2b, c). In the presence of berberine (5 Equivalents) the T_m_ was increased to ~85 °C (Fig. 2b, c), indicating that the RET G-quadruplex could be stabilized with berberine.

### Selective suppression of RET in TT cells by berberine

**Fig. 3:**
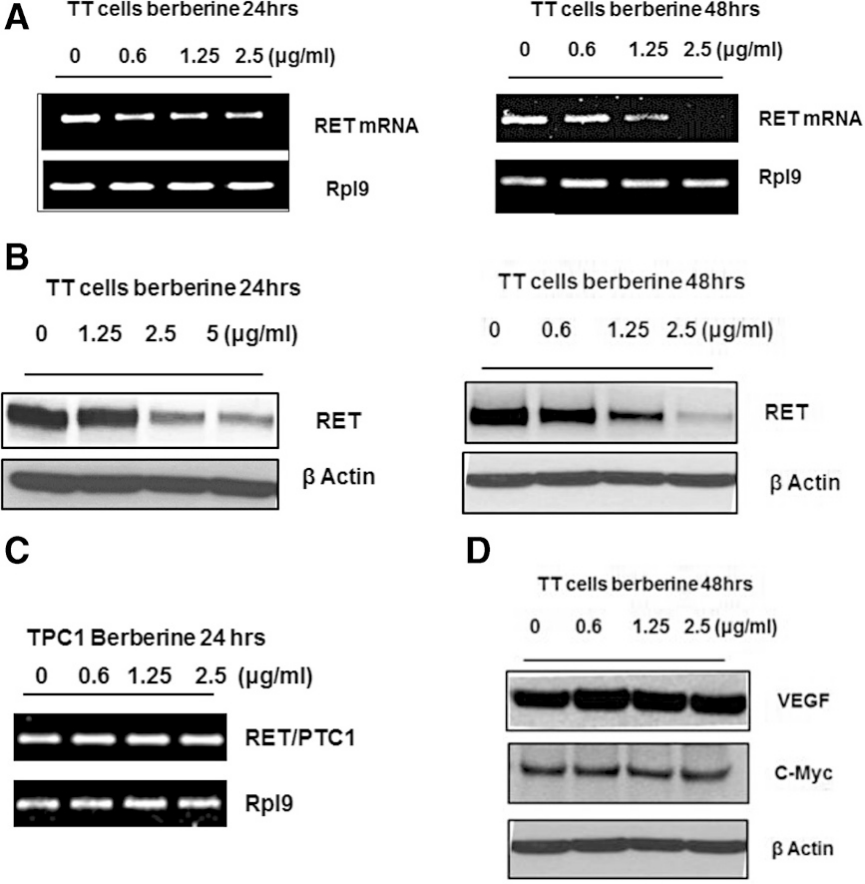
Effect of berberine on *RET* expression in both the TT and TPC1 cell lines. **a** Effect of berberine on the RET mRNA expression in TT cells after 24 and 48 h treatments at various concentrations. **b** RET protein expression in TT cells were determined by western blotting after the 24 and 48 h exposure to increasing concentrations of berberine. **c** Effect of berberine on the RET/PTC1 mRNA expression in TPC1 cells was determined following 24 h exposure with various concentrations of berberine. **d** Protein expression levels of VEGF and c-MYC in TT cells in the presence of berberine at various concentrations and exposed up to 48 h

Since our *in vitro* studies revealed that berberine is a potential small molecule that interacts with and stabilizes the G-quadruplex structure formed on the *RET* promoter region, we next examined the effect of berberine on the *RET* expression using *in vitro* cell-based assays. The MTC TT cell line was chosen in our study because the expression of the *RET* proto-oncogene in this cell line is under the control of the promoter region that contains the G-rich sequence, which adopts G-quadruplex structure [19]. As shown in Fig. 3a, berberine decreased the *RET* gene expression by more than 50 % and 90 % after 24 and 48 h exposure respectively at a non-toxic concentration of 2.5 μg/ml. The RET protein expression was also diminished based on the western blot analysis (Fig. 3b).

To further probe whether the G-quadruplex structure formed in the promoter region of *RET* gene is the intra-cellular target for berberine, papillary thyroid carcinoma (PTC) TPC1 cells were used. The *RET/PTC1* gene expression in this cell line is under the control of the promoter region of the *CCD6* gene due to chromosomal rearrangement [36–39], which lacks the G-quadruplex forming motif. As shown in the Fig. 3c the RET/PTC1 mRNA expression was not significantly decreased in the presence of berberine following 24 h exposure. This data clearly suggests that the presence of G-quadruplex forming sequence on the proximal promoter region of the *RET* gene, plays a critical role in mediating the effect of berberine on gene expression.

### Effect of berberine on *RET* promoter activity

**Fig. 4:**
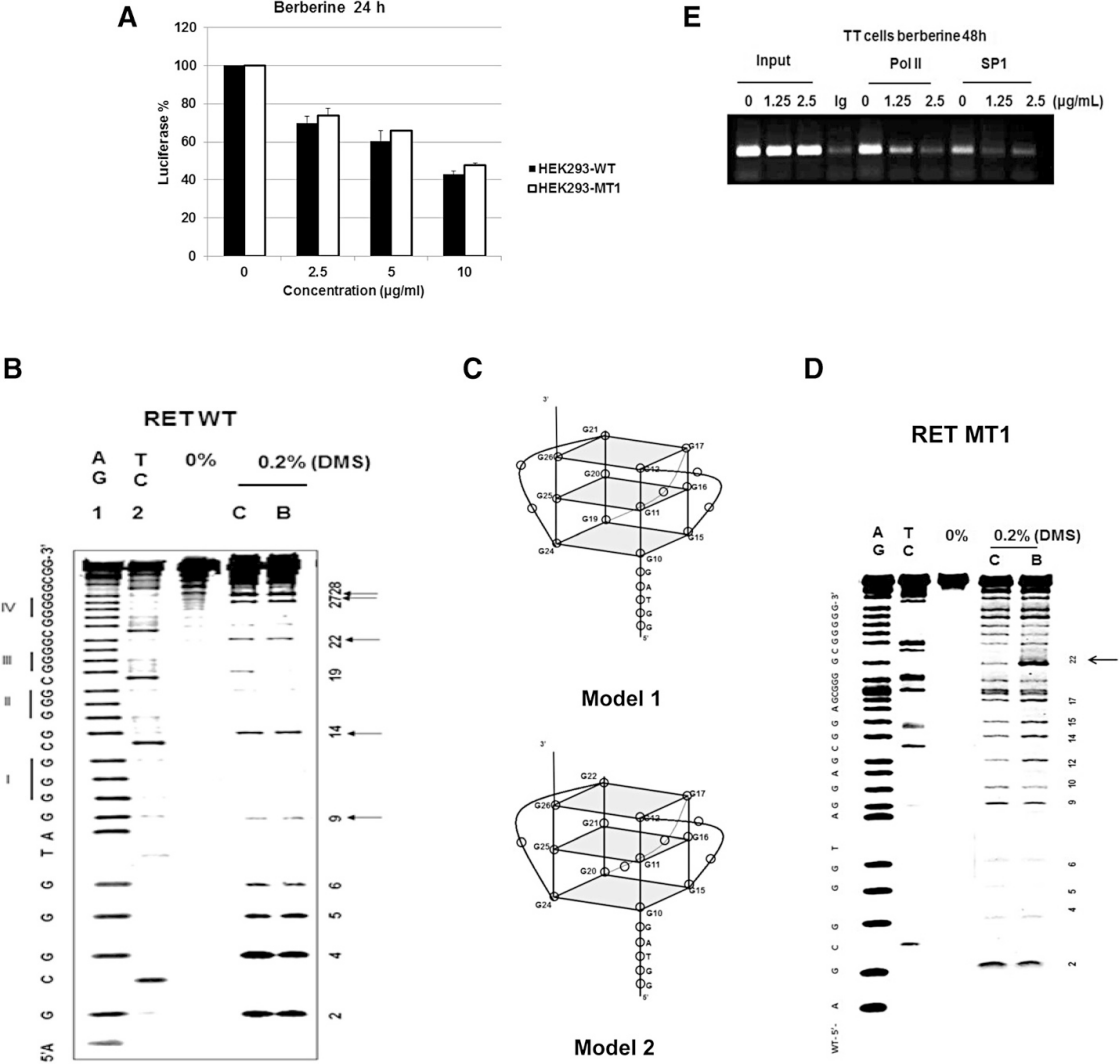
Effect of berberine on the promoter activity of the *RET* gene. **a** Effect of berberine on the luciferase expression in HEK293-WT and HEK293-MT1 cell lines following the treatment with berberine up to 24 h. Luciferase activity in cell lysates was measured as relative luminescence units (RLU) and normalized to the total protein content. Experiments were performed in triplicate. Error bar represent one s.d. above and below the mean % luciferase activity. **b** DMS foot printing on the RET-WT G-quadruplex forming sequence in the absence and in the presence of berberine (5 Equivalents) following 0.2 % DMS treatment (lanes C & B respectively). Purine & pyrimidine sequencing act as single base ladders to identify the protected and cleaved guanines after piperidine treatment (lanes 1 & 2 respectively). **c** Schematic models for the parallel G-quadruplexes formed by RET-WT (**d**) DMS foot printing on the RET-MT1 quadruplex forming sequence in the absence and in the presence of 5 equivalents of berberine following 0.2 % DMS treatment (lanes C & B respectively). **e** ChIP analysis to determine the effect of berberine in recruiting the SP1 and RNA pol II to the GC box region in the RET promoter region in TT cells following 48 h exposure at two different concentrations. Recruitment of SP1 and Pol II to the RET proximal promoter region was assessed by PCR using RET promoter specific primers. 1 % of the input DNA was used as internal control (Input) and isotype-matched IgG was used as a negative control for immunoprecipitation (Ig)

To investigate whether berberine suppresses the *RET* gene expression by inhibiting its promoter activity through G-quadruplex stabilization, two isogenic cell lines HEK293-WT and HEK293-MT1 in which the expression of the luciferase reporter gene is under the control of wild-type and G4 knockout (KO) mutant *RET* promoter regions respectively were used [19]. As shown in Fig. 4a, approximately 60 % decrease in the luciferase expression was observed in both cell lines in the presence of berberine (10 μg/ml). This suggests that the inhibitory effect of berberine is independent of the G-quadruplex structure formed on the *RET* promoter region because the G4 KO sequence in the HEK293-MT1 cell line is unable to form this structure [19]. Based on this data we predicted that berberine could be able to bind the RET-MT1 sequence on the promoter region and might form an unknown structure, through which it inhibits *RET* gene promoter activity.

To verify our prediction, we investigated the mode of interaction of berberine with both RET-WT & RET-MT1 G-rich sequences by DMS footprinting. DMS footprinting is a well-known technique to determine the guanine bases that are involved in the G-quadruplex formation. The N7 positions of these guanines are involved in Hoogsteen base-pairing to form the G-tetrad and are protected from methylation by DMS [25, 40, 41]. As shown in Fig. 4b lane C, the pattern of N7-guanine methylation produced by the RET WT sequence in the presence of 100 mM K^+^ is consistent with two intra-molecular parallel G-quadruplexes each containing three G-tetrads (Fig. 4c, Model 1&2). As shown in Fig. 4b lane B, after the addition of berberine (5 Equivalents), the pattern of N7-guanine methylation is consistent with a single intra-molecular parallel G-quadruplex as shown in the Fig. 4c (Model 1) suggesting that the binding of berberine with the G-quadruplexes transforms them into a single conformation. Based on the DMS footprinting on the RET-MT1 sequence in the absence and presence of berberine (5 Equivalents), the guanine G22 in the G-quadruplex/berberine complex (Fig. 4d lane B) clearly showed an enhanced cleavage compared to the unbound G-quadruplex (Fig. 4d lane C). This clearly indicates the interaction of berberine with the G4 knockout (KO) mutant sequence on the *RET* promoter region.

To further understand the cellular mechanism through which berberine inhibits the *RET* expression, a Chromatin Immunoprecipitation (ChIP) assay was performed to investigate the effect of berberine on the *RET* promoter occupancy by SP1 and RNA pol II in TT cells. As shown in Fig. 4e, the ChIP assay revealed that berberine prevents the SP1 and pol II binding to the RET promoter, suggesting that the G-quadruplex interacting agents might possibly interfere with the transcription complex assembly at the *RET* promoter region.

### Effect of canadine on the RET promoter activity

**Fig. 5:**
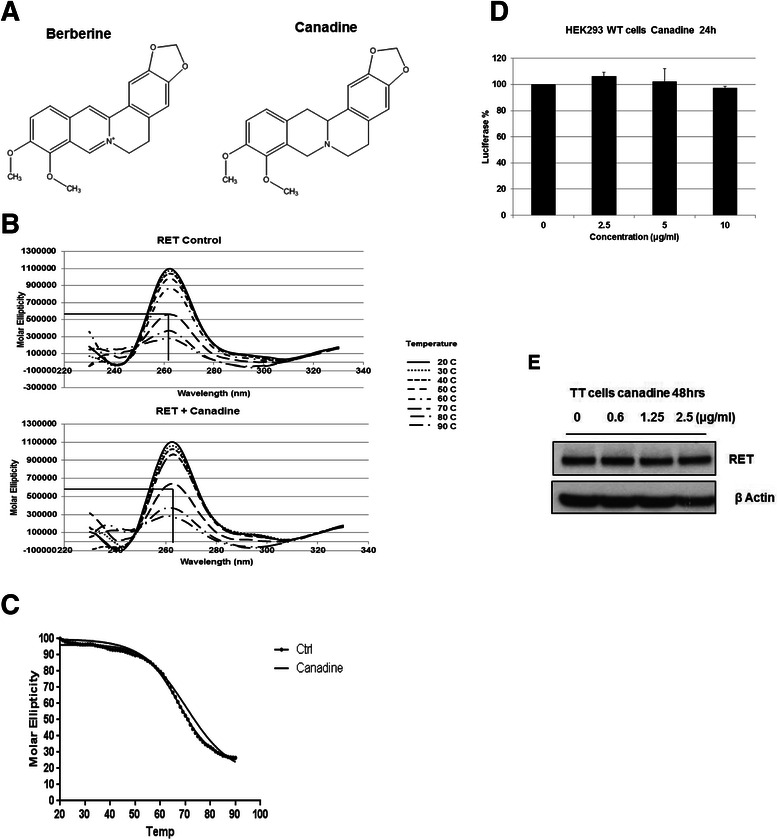
Effect of canadine on the G-quadruplex stabilization and on the RET promoter activity. **a** Structures of berberine and canadine. **b** CD titration spectra for the RET-WT (5 μM) in the absence and in the presence of canadine (5 Equivalents) with increasing temperatures from 20 °C to 90 °C. **c** Melting curve of the RET-WT G-quadruplex in the absence and in the presence of canadine (5 Equivalents). **d** Effect of canadine on the luciferase expression level in HEK293-WT cell line at various concentrations after 24 h treatment. **e** Effect on the RET protein expression in TT cells after 48 h incubation at an increasing concentrations of canadine

Canadine is one of the analogues, which closely resembles the structure of berberine [42]. However the saturated double bonds in the isoquinoline ring might alter its aromaticity [42], thereby interrupting it’s binding with the G-quadruplex (Fig. 5a). The CD spectra of the RET G-quadruplex at increasing temperatures were obtained in the absence and presence of canadine (5 Equivalents). As anticipated the T_m_ of the RET G-quadruplex structure was not significantly increased after the addition of canadine (5 Equivalents) (Fig. 5b, c), suggesting that canadine does not stabilize the G-quadruplex structure formed in the RET promoter region.

Next, we investigated if canadine has differential effect from berberine on the *RET* gene promoter activity by measuring the luciferase expression level in the HEK293-WT cells in the presence of various concentrations of canadine. As shown in Fig. 5d, no significant decrease in the luciferase level was observed even at 10 μg/ml following 24 h exposure. The RET protein expression level in TT cells was also not decreased after 48 h treatment with canadine (Fig. 5e). Overall, these data suggest that the stabilization of the G-quadruplex structure in the RET promoter region with berberine is important in suppressing the gene transcription.

### Cellular effects mediated by the RET expression

**Fig. 6:**
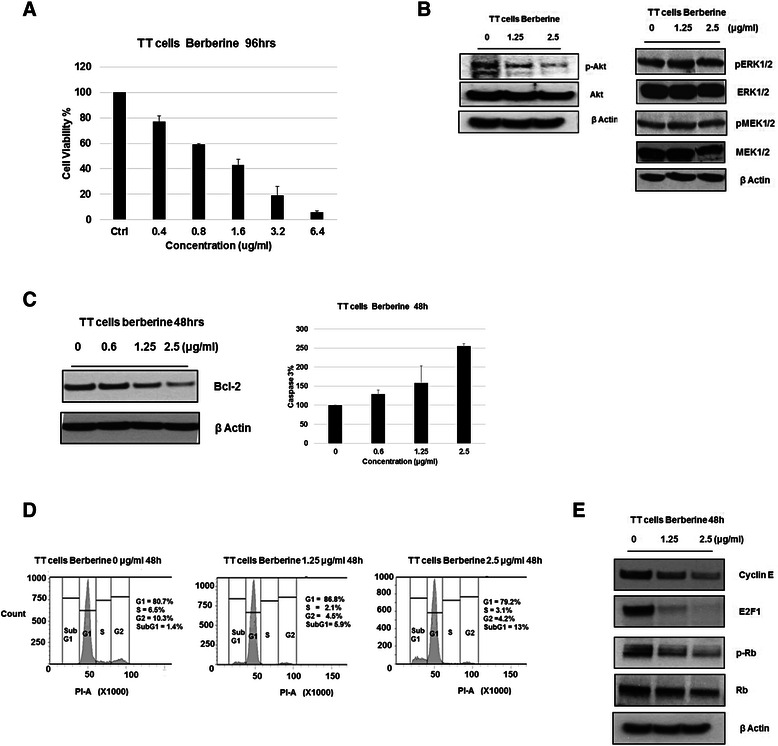
Cellular effects mediated by RET expression in TT cells. **a** MTT assay for TT cells, treated with an increasing concentration of berberine for 96 h to determine the cell viability. **b** The phosphorylation status of Akt, MEK1/2 and ERK1/2 were determined in TT cells following the exposure with different concentrations of berberine. **c** The Bcl-2 and the Capsase-3 activity in TT cells were determined in the presence of increasing concentrations of berberine. **d** FACS analysis of TT cells treated with various concentrations of berberine for 48 h. Data are mean +/−s.e.m of three separate experiments. **e** Western Blot analysis to determine the protein expression of pRb, p-pRb, E2F1, Cyclin E in TT cells

Since RET is a growth factor receptor and mainly involved in mediating the cell proliferation and viability, we examined the effect of berberine on the growth of TT cell line by MTT assay. As shown in Fig. 6a, the viability of TT cells decreased with increasing concentrations of berberine and the IC_50_ was found to be approximately 1 μg/ml after 96 h exposure. This clearly shows that the *RET* expression plays a major role in regulating the cell proliferation and viability.

We further characterized the mechanism through which berberine suppresses the proliferation of TT cells by determining whether the mitogenic signaling pathways of RET are inhibited. Following the exposure of TT cells with berberine, the phosphorylation status of MEK/ERK and Akt were determined, as they are the major kinases involved in the signal transduction pathways activated by oncogenic RET [9]. As shown in the Fig. 6b, berberine inhibits the phosphorylation of Akt in a dose-dependent manner whereas the phosphorylated MEK/ERK levels were not altered. This suggests that the inhibition of *RET* expression by berberine in TT cells results in the down-regulation of the PI3k/Akt pathway.

Next, we investigated how the reduced levels of phosphorylated Akt contribute to the suppression of cell proliferation by determining the effect of berberine on the downstream targets of this pathway. Based on the previous reports the PI3K/Akt pro-survival pathway activates the anti-apoptotic Bcl-2, thereby preventing the caspase-3 activation [9]. Interestingly, berberine reduced the Bcl-2 expression in TT cells, which in turn showed 2.5 fold increase in the caspase-3 activity compared to the untreated cells (Fig. 6c). Flow cytometry analysis of the TT cells after 48 h incubation with different concentrations of berberine showed an increase in the percentage of cells at G1 phase at 1.25 μg/ml, indicating G1 arrest (Fig. 6d). At high concentration of berberine (2.5 μg/ml) increase in apoptotic cells was observed, which was represented by a higher percentage of TT cells in the sub-G1 phase as compared to the untreated cells (Fig. 6d). The increase in apoptosis is further accompanied by decrease in the percentage of cells at both S and G2 phases, which clearly indicates that berberine induces apoptosis in TT cells through G1-arrest (Fig. 6d). To further confirm the cell cycle-arrest mediated by berberine, the expression of cell-cycle regulatory proteins were determined. The phosphorylation of the retinoblastoma protein Rb at the G1 phase releases the transcription factor E2F1, which in-turn transactivates cyclin E that is required to progress through S phase [43]. As shown in the Fig. 6e, the expression of the phosphorylated Rb, E2F1 and cyclin E were reduced significantly in the presence of berberine, which clearly indicates the cell cycle arrest.

## Discussion

The main objective of our study is to highlight a therapeutic strategy to specifically treat MTC by silencing the transcription of *RET* gene using small molecules. Although the use of small interfering RNAs (siRNAs) has been developed as a promising strategy to silence the gene expression [44], there are several pitfalls in developing siRNAs as drug-like molecules. The siRNAs are negatively- charged and large in size that thwart their permeability into the plasma membrane of the target cells and also they are highly susceptible to degradation by the enzymes present in the extracellular region, thereby decreasing its therapeutic potential [45]. Hence, the use of small-molecule inhibitors that diffuse readily into the cell membranes and suppress the gene expression overcomes these pitfalls and challenges. The transcriptional inhibitory effect exerted by the formation of the G-quadruplex structure on the *RET* gene promoter region led us to stabilize this secondary structure using small molecules to suppress the gene expression. Based on the results obtained from *in vitro* biochemical and cell-based assays, an isoquinoline alkaloid called berberine has been identified as a potential small-molecule to bind and stabilize the RET G-quadruplex. The stabilization of the G-quadruplex structure by berberine also exerted inhibitory effects on the RET promoter activity, which was observed by the luciferase expression that is under the control of RET promoter region. The ChIP assay further revealed that berberine prevented the binding of SP1 and PolII with the RET promoter region thereby inhibiting the transcription of this gene. Overall, our data suggest that the cellular target for berberine might be the G-quadruplex structure formed on the *RET* promoter region.

The most challenging issue in the target based drug discovery is to address the specific interaction of a compound with its target. Hence it is important to demonstrate that the formation of the G-quadruplex structure on the proximal promoter region of the *RET* gene is strictly required to mediate the inhibitory effect of berberine. To address this issue, we included the papillary thyroid carcinoma (PTC) TPC1 cells in our study. Like MTC, the development of PTC is also due to the constitutive activation of the RET protein in a ligand independent manner [36]. However, the gain-of-function mutation of this protein in PTC is due to chromosomal rearrangement of the *RET* kinase domain coding region with the 5′ terminal of the coiled-coil domain containing gene 6 (*CCD6*) at chromosome 10q11.2 [36]. This results in a truncated fusion protein called RET/PTC1 in which the dimerization domain of the *CCD6* gene mediates the formation of RET homo-dimers, followed by phosphorylation of the tyrosine residues and activation of the downstream signaling pathways [38]. Due to the chromosomal inversion the *RET/PTC* gene is under the control of CCD6 promoter region, which does not have the GC box region and thus cannot form the G-quadruplex structure. In our study, the *RET/PTC1* mRNA levels were not decreased in the presence of berberine, suggesting that this compound is highly specific to MTC due to the G-quadruplex forming sequence present on the *RET* promoter. Although, the previous study has demonstrated the anti-proliferative effects of berberine in TPC1 cells, the transcriptional inhibitory effect of berberine on the *RET/PTC1* gene expression was not addressed [46].

To further confirm the specificity of berberine in targeting the G-quadruplex structure, we took advantage of the structure-activity relationship that mediates the binding with this structure. Canadine resembles the structure of berberine but it has a tertiary amine group due to the saturation of the double bonds in the isoquinoline ring, which alters its planarity due to the loss of π electrons [42]. This disrupts the binding potential of canadine with the G-quadruplex thereby exhibiting no biological activity in the TT cells. This shows that the inhibition of the *RET* gene in TT cells by berberine is only due to its interaction with and stabilization of the G-quadruplex structure.

In our study, berberine showed a selective effect on the *RET* gene expression by inhibiting its transcription, without significantly affecting the other major oncogenes like *c-Myc and VEGF* (Fig. 3d), which also have the GC-rich sequence on their promoter regions [40, 41]. A plausible explanation is that the intra-cellular G-quadruplex formation is more predominant in the *RET* promoter region than the other regions in the chromosome, which favors the specific interaction of berberine. Even though berberine has been shown to suppress c-MYC expression [47], the concentration at which it is active against this oncogene is several fold higher than that against the RET expression. We have also shown that the disruption of the oncogenic RET expression in TT cells in the presence of berberine further suppressed the cell proliferation and viability through the down-regulation of PI3K/Akt pathway, which in turn activates apoptotic cell death. The MEK/ERK pathway, which has also been known as the downstream signal transduction pathway was not inhibited by berberine suggesting that either this pathway could be governed by other growth factor receptors like VEGFR, EGFR etc. [9, 48, 49] or prolonged exposure with berberine could inhibit this pathway.

## Conclusion

We have clearly demonstrated that berberine selectively suppresses the transcription of *RET* proto-oncogene through the stabilization of the G-quadruplex structure formed on the promoter region. The downstream signaling pathways that are mediated by RET activation were also down-regulated in the presence of berberine, which further inhibited the MTC TT cell proliferation through cell cycle arrest and the activation of apoptosis. Hence berberine has definite potential as a drug, based on its diverse pharmacological properties and activities, which could be further developed for the pre-clinical and clinical studies to treat MTC patients and also be used in combination therapy with other RET kinase inhibitors.

## Abbreviations

MTC: Medullary Thyroid Carcinoma
PTC: Papillary Thyroid Carcinoma
DMS: Dimethyl Sulfate
ChIP: Chromatin Immuno-precipitation
CCD6: Coiled-coil Domain containing gene 6
